# Propensity Score Matching Analysis of Differential Outcomes in Holmium Laser Enucleation of the Prostate vs. Robotic-Assisted Simple Prostatectomy

**DOI:** 10.3390/jcm13175135

**Published:** 2024-08-29

**Authors:** Narmina Khanmammadova, James F. Jiang, Ralph Kevin Medina Gomez, Ashley Gao, Timothy Young Chu, Mohammed Shahait, Kristene Myklak, David I. Lee, Akhil K. Das

**Affiliations:** 1 Department of Urology, University of California Irvine, 3800 W Chapman Ave., Suite 7200, Orange, CA 92868, USA; nkhanmam@hs.uci.edu (N.K.);; 2 School of Medicine, University of Sharjah, Sharjah 26666, United Arab Emirates

**Keywords:** benign prostatic hyperplasia, Holmium laser enucleation of the prostate, robot-assisted simple prostatectomy, propensity score matching

## Abstract

**Background & Objectives:** Patients with bladder outlet obstruction (BOO) due to massive prostate enlargement have several surgical treatment options, such as robot-assisted simple prostatectomy (RASP) and holmium laser enucleation of the prostate (HoLEP). Postoperative outcomes may differ between those undergoing RASP and HoLEP. RASP has been associated with a lower incidence of transient stress urinary incontinence (SUI), while HoLEP allows for shorter catheterization times. Here, we report on our experience with both surgical modalities. **Methods:** Data were collected from prospectively maintained databases for 37 RASP patients and 181 HoLEP patients treated from July 2021 to November 2023. To control for selection bias, propensity score matching (PSM) was utilized based on age and prostate size. We compared patients’ preoperative, perioperative, and postoperative outcomes both before and after applying PSM. **Results:** Before the PSM, the median prostate size was significantly lower in the HoLEP group (*p* < 0.001). The HoLEP group also had significantly shorter operative times (*p* ≤ 0.001) and lower weights of resected adenoma (*p* ≤ 0.001). After the PSM of 31 RASP and 31 HoLEP patients, all baseline patient characteristics were comparable. No significant differences were observed in operation time (*p* = 0.140) or in the weight of resected adenoma (*p* = 0.394) between the modalities. The median (IQR) length of catheterization was significantly shorter in the HoLEP group (1 [1–4] days) compared to the RASP group (7 [7–8] days), in both pre- and post-matching analyses (*p* ≤ 0.001 for both), reflecting the standard of practice. In contrast, in both pre- and post-PSM analyses, the average hospital stay was significantly shorter in the RASP cohort, as same-day discharge is standard in our center, whereas the HoLEP cohort required overnight stays due to routine continuous bladder irrigation before discharge (*p* < 0.001 for all). Notably, the SUI rates and American Urological Association (AUA) symptom scores were comparable at 3 months within both matched and unmatched cohorts (pre-PSM: *p* = 0.668, *p* = 0.083; post-PSM: *p* = 1, *p* = 0.152, respectively). **Conclusions:** Our comparative analysis indicates that both RASP and HoLEP yield similar outcomes, including SUI rates, at 3 months. While HoLEP provided shorter durations of postoperative catheterization, RASP offered shorter hospital stays.

## 1. Introduction

Benign prostatic hyperplasia (BPH) is an extremely common condition affecting many older men, with almost 80% of men over the age of 70 affected [1]. Historically, transurethral resection of the prostate (TURP) has been the gold standard for the treatment of small to medium-sized prostates. However, TURP is not as efficacious for the treatment of large glands ≥80 g and is associated with higher rates of complications such as longer operative time, increased bleeding, and higher risk of re-operation and transfusion [2]. Open simple prostatectomy (OSP) was another treatment of choice for large glands (≥80 g) [2]. However, during the last few decades, Holmium laser enucleation of the prostate (HoLEP) and robotic-assisted simple prostatectomy (RASP) have emerged as efficacious minimally invasive treatment options for glands ≥80 g, with both allowing for complete removal of the prostatic adenoma.

HoLEP and RASP offer several advantages compared to other surgical modalities for treating BPH. By removing the entire transition zone of the prostate, HoLEP has demonstrated excellent durability for long-term relief of BPH symptoms and significantly lower re-treatment rates of 0–1.4% at 7–10 years. This is in contrast to 17.7% following TURP and 28.9% following prostatic urethral lift (PUL) [3,4]. Furthermore, HoLEP is more efficacious than TURP, with improved outcomes such as better hemostasis, and short-term urinary parameters, fewer immediate complications, reduced catheter times, and shorter hospital stays [5,6]. Similarly, RASP has been found to achieve comparable voiding outcomes relative to OSP, while also providing reduced blood loss, transfusion rates, and length of hospitalization [7].

HoLEP and RASP have different post-operative recovery courses and side effects that may influence a patient’s choice of treatment modality. HoLEP not only provides the unique benefits of being a minimally invasive endoscopic procedure independent of prostate size but also is endorsed under the American Urological Association (AUA) Guidelines for patients on blood thinners. Previously, a few studies comparing these two treatment modalities have shown that, compared to RASP, HoLEP results in minimal bleeding with lower transfusion rates, shortened operative and catheter times, and reduced lengths of hospital stay [8,9,10]. On the other hand, RASP offers benefits such as a shorter learning curve and lower rates (2%) of post-operative de novo transient stress urinary incontinence (tSUI) compared to HoLEP (11%, *p* < 0.01) during the early recovery period [10]. Although tSUI is expected post-HoLEP, with the newer early apical release techniques, rates as low as 5.8% at 1 month and 0.7% at 6 months have recently been reported [11].

In this study, we report the comparative outcomes of both surgical modalities at a single institution. To the best of our knowledge, this is the first analysis based on propensity score matching of RASP with a bladder neck sparing technique and HoLEP with the early apical release technique.

## 2. Materials and Methods

### 2.1. Data Collection and Patient Population

From July 2021 to November 2023, 181 patients underwent HoLEP, and 37 patients underwent RASP at our center. After the Institutional Review Board (IRB) approval, data were queried from two prospectively maintained databases. All cases were performed by two surgeons: HoLEP by A.K.D. and RASP by D.I.L. Both surgeons are very experienced with their modalities.

**Figure 1:**
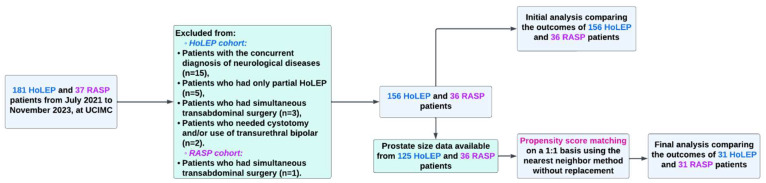
Study Flowchart.

Patients with the following characteristics were excluded: concurrent neurologic disease (n = 15), only partial HoLEP (n = 5), simultaneous transabdominal surgery (n = 4), required cystotomy, and/or the use of transurethral bipolar (n = 2). This left 156 HoLEP and 36 RASP patients for the final analysis (Figure 1).

Preoperative data included age, body mass index (BMI), prostate size (US or MRI or CT scan), previous interventions for BPH, use of alpha-blockers and/or 5-alpha reductase inhibitors (5-ARIs), history of urinary retention, AUA Symptom Score (AUASS), and postvoid residual volume (PVR). Perioperatively, we recorded the American Society of Anesthesiologists Physical Status Classification System (ASA) score, operation time, estimated blood loss (EBL), complications, catheterization duration, and length of hospital stay. Postoperative data encompassed complication rates, prostate-specific antigen (PSA) levels, 90-day readmission rates, pathological assessments of the specimen—including tissue type and weight of the resected adenoma reported on pathology results, PVR, AUASS, and SUI rates at 3 months.

SUI was assessed during postoperative follow-up visits. Self-administered AUASS surveys were distributed during clinic visits or electronically via Research Electronic Data Capture (REDCap Version 14.6.2, Vanderbilt University, Nashville, TN, USA) software [12]. Complications were classified using the Clavien-Dindo grading system [13].

### 2.2. Statistical Analysis

Categorical variables are presented as frequencies and proportions, whereas numerical data are reported as means with standard deviations (SD) or medians with interquartile ranges (IQR). For continuous variables, the independent sample *t*-test or Mann-Whitney U test was used as appropriate. Fisher’s exact test or Pearson Chi-square test was utilized for categorical variables when applicable. Additionally, univariate logistic regression analyses were conducted to evaluate the predictive ability of the treatment option on various outcomes, including postoperative SUI rates, AUASS at 3 months, and complication rates. Two-sided *p*-values were reported, with a *p*-value < 0.05 indicating significance. IBM SPSS Version 28 (IBM Corp., Armonk, NY, USA) was used for analysis.

To minimize selection bias, propensity score matching (PSM) was applied. A propensity score is the likelihood of a subject receiving a specific covariate, determined by the distribution of associated variables. In this study, PS were calculated using logistic regression, considering age and prostate size. Data on prostate size were available from 125 of the 156 HoLEP patients (80.1%) and all 36 RASP patients (100%), who were included in the matching. We performed manual matching using a 1:1 nearest neighbor method without replacement, setting the caliper width at 0.2 times the SD of the logit of the PS. We compared preoperative, perioperative, and postoperative outcomes before and after PSM. Initially, outcomes from 156 HoLEP and 36 RASP patients were analyzed. Post-PSM, the analysis included 31 patients from each group.

### 2.3. Surgical Techniques

HoLEP cases were performed utilizing the top-down technique with an early apical release by a single surgeon (A.K.D.) as previously described by Hodhod et al. [14]. At our institution, we use a 26 French (Fr) continuous flow resectoscope with a laser bridge and endoscopic camera (Karl Storz, Tuttlingen, Germany). The inflow port is connected to room temperature normal saline irrigation at 60 mmHg and the outflow port is left to gravity drainage. A 7.1 Fr tapered open-ended laser ureteral catheter (Cook Medical, Bloomington, IN, USA) with a silicone membrane adaptor at the end is passed through the laser bridge which stabilizes the laser at the 6 o’clock position of the endoscope. We use a single-use 550-micron end-firing Holmium: YAG laser fiber (Lumenis Pulse^TM^ Holmium Laser System with MOSES^TM^ 2.0 Technology, Lumenis, San Jose, CA, USA) with an energy level of 2.0 J and frequency of 30–50 Hz for both incision and coagulation. A soft tissue morcellator (PIRANHA Enucleation System, Richard Wolf, Knittlingen, Germany) is used. All patients are then admitted for continuous bladder irrigation (CBI) and given a voiding trial on postoperative day one; the majority are discharged without a catheter in place.

RASP was performed utilizing the bladder neck sparing technique by a single surgeon (D.I.L.) as previously described by Shahait et al. [15]. Via a transperitoneal approach, the preperitoneal space is developed. The bladder neck dissection is performed to completely separate the bladder from the prostate similar to a robotic radical prostatectomy. The prostatic adenoma is separated from the surgical capsule with a combination of blunt dissection and electrocautery. A circumferential 360° anastomosis closure as described by Van Velthoven, using a running, double-armed 3–0 bidirectional absorbable barbed Quill™ (Ethicon Inc., Somerville, NJ, USA) suture is performed [16]. An 18 F two-way foley catheter is placed and tested to ensure a watertight closure. No CBI is utilized. Finally, a robot-assisted transperitoneal transversus abdominis plane (TAP) block is performed as previously described [17]. Patients are observed for a short period in the post-anesthesia care unit before same-day discharge (SDD). The patient returns to a clinic for a voiding trial in 7 days. 

Of note, at our center, all patients undergoing either HoLEP or RASP are strongly encouraged to start pelvic floor strengthening exercises during the early postoperative period.

## 3. Results

### 3.1. Before PSM

The initial analysis included 156 HoLEP patients and 36 RASP patients (Table 1). Before PSM, the median (IQR) prostate size for HoLEP was significantly smaller when compared to the RASP group (84 (54.5–120) vs. 141.5 (104–158), respectively; *p* < 0.001). All other baseline cohort characteristics were comparable between the two groups, including age, BMI, rates of previous BPH interventions, use of medications, history of retention, PVR volume, and AUASS (*p* > 0.05 for all). Perioperatively, the HoLEP group had significantly shorter operation time (*p* < 0.001), and length of catheterization (*p* < 0.001), as well as lower EBL (*p* < 0.001). In contrast, the RASP cohort had a significantly shorter length of hospital stay (*p* < 0.001) and a higher weight of resected adenoma (*p* < 0.001). We did not observe any differences in perioperative and postoperative complication rates, 90-day readmission rates, distribution of ASA scores, postop PSA levels, and PVR volumes. Similarly, postoperative AUASS and SUI rates at 3 months were comparable between the two cohorts.

### 3.2. After PSM

Following PSM, 31 RASP and 31 HoLEP patients were included in the final analysis and all baseline patient characteristics were comparable between the two groups (Table 2). The mean ± SD age was 70 ± 8.1 years for the HoLEP group and 71 ± 8.2 years for the RASP cohort. After PSM, the median (IQR) prostate size was comparable between the two groups (117 (95–160) for HoLEP vs. 134 (103–154) for RASP; *p* = 0.335). The rates of preoperative urinary retention (58.1% HoLEP vs. 51.6% RASP, *p* = 0.799) and average preoperative AUASS (21 ± 9 for HoLEP vs. 20 ± 6 for RASP, *p* = 0.707) were similar. 

Post-PSM, no difference was observed in operation time (*p* = 0.140) or postoperative complication and readmission rates (*p* = 0.707, *p* = 1, respectively) between modalities. The reasons for hospital readmission in both cohorts are detailed in Table 3. Both groups had a median EBL of equal to or less than 100 cc (40 (20–50) for HoLEP and 100 (50–100) for RASP, *p* < 0.001). Although there is a statistically significant difference in EBL, no complications related to blood loss were experienced by any patient in the RASP group. The mean (±SD) pathological weight of resected adenoma was comparable (78.5 ± 36.4 for HoLEP, 70.9 ± 32 for RASP, *p* = 0.394). The fractional prostate weight resected was 67.1% for HoLEP and 53% for RASP, respectively. Additionally, the average post-operative PSA for both groups was 0.8 ng/mL (*p* = 0.879). The median (IQR) length of catheterization was significantly shorter in the HoLEP group when compared to the RASP group (1 (1–4) vs. 7 (7–8), respectively; *p* < 0.001). However, the median (IQR) length of hospital stay was significantly shorter in the RASP cohort in contrast to the HoLEP group (*p* < 0.001). SUI rates at 3 months were comparable, with a rate of 7% for HoLEP and 8% for RASP (*p* = 1). AUASS scores at 3 months were also comparable; 94% of HoLEP patients and 73% of RASP patients reported scores ≤ 7 (*p* = 0.152).

In univariate regression analyses evaluating the effects of surgical interventions on outcomes, including SUI rates, AUASS at 3 months, and complication rates, no statistically significant associations were detected in either the pre- or post-PSM matching cohorts (*p* > 0.05 for all) (Table 4). Consequently, constructing multivariable models to predict these outcomes was not feasible.

## 4. Discussion

In this study, we present the first PSM-based analysis of comparative surgical outcomes following RASP with a bladder neck sparing technique and HoLEP with early apical release. As prior studies have demonstrated varying outcomes between RASP and HoLEP [8,9,10], our study contributes three major findings to the literature. First, both techniques yielded favorable functional outcomes for the surgical management of BPH, demonstrating comparable amounts of prostatic adenoma removal and operative times after PSM, considering prostate size (*p* > 0.05 for both). Second, we show that similarly low rates of tSUI are achievable following both procedures. Third, HoLEP had a shorter average catheter time of 1 day, compared to 7 days for RASP whereas RASP provided shorter hospital stays (*p* < 0.001, for both).

Here we report that, following PSM that considered prostate size, both RASP and HoLEP resulted in comparable amounts of tissue removed and similar post-operative AUASS scores at 3 months. Both procedures involved the complete removal of the prostatic adenoma, evidenced by high weights of adenoma removed and low average post-operative PSA levels (0.8 ng/mL for both modalities, *p* = 0.879).

Previously, Zhang et al. reported that HoLEP had shorter operative times compared to RASP [9]. Initially, in our study, the median (IQR) operation time was significantly shorter in the HoLEP group (109.5 [77–134]) when compared to the RASP group (131 [116.25–144.25]; *p* < 0.001). However, this could be explained by the significantly smaller average prostate size in the HoLEP cohort (*p* < 0.001). Following 1:1 PSM based on age and prostate size, there was no significant difference in the mean (±SD) operative time between the two modalities (*p* = 0.140). These results suggest that when prostate size is considered, the average operation time is comparable between HoLEP and RASP. In addition, differences in perioperative outcomes among different studies can primarily be attributed to two factors: surgeon expertise and variations in surgeon-specific standard practices. In our study, both surgeons are high-volume experts in RASP and HoLEP with over 20 years of experience performing and teaching these surgeries, ensuring that in experienced hands, RASP can be completed with an operative time comparable to HoLEP, as similarly reported by others [10].

Historically, compared to RASP, HoLEP had more concerns of postoperative tSUI attributed to the strain on the external urethral sphincter during retrograde enucleation of the prostatic adenoma. Multiple studies have shown that the incidence of tSUI following HoLEP ranges from 10% to 44%, depending on technique and surgeon experience [10,18,19]. While some studies [20] found no differences in post-operative tSUI between HoLEP and RASP, others have reported tSUI rates at 3 months of 28% for HoLEP compared to 5% for SP-RASP [10]. This higher rate was attributed to several factors: the cases being completed during the early learning curve of the surgeon, HoLEP being new to the institution, and the early apical release technique not yet being adopted [10]. With newer early apical release techniques, tSUI rates have been reported at 5.8% at 1 month and 0.7% at 6 months [11]. In our study, we demonstrated comparable rates of postoperative SUI at 3 months between HoLEP and RASP in patients with large prostates (7% vs. 8%, respectively, *p* = 1). We attribute this to the use of the early apical release technique during HoLEP, early post-operative pelvic floor strengthening for both modalities, as well as surgeon and institutional experience in RASP and HoLEP.

It has been shown that RASP has concerns related to longer postoperative catheter times. Like other studies [8,9,10], we demonstrate a significantly lower average postoperative catheter time for HoLEP (1 day) compared to RASP (7 days, *p* < 0.001). Following RASP, a postoperative catheter length of 7 days is standard in our institution to allow bladder healing and prevent bladder leaks. Of note, the development of single-port RASP (SP-RASP) with a transvesical approach has led to reports of decreased postoperative catheter times. SP transvesical RASP utilizes a single 3.5 cm skin incision and extraperitoneal access, eliminating the need for steep Trendelenburg, pneumoperitoneum, and bowel manipulation [10]. This smaller cystotomy used in SP-RASP has prompted some practices to adopt routine catheter removal on postoperative day 2 [10]. These results are promising for the progression of RASP and the improvement of postoperative recovery and catheter times. However, these reports are from high-volume expert single-port robotic surgeons, and the widespread adoption of these techniques is yet to be seen. The costs and high learning curve associated with SP robotics are well-documented, which may limit the expanded use of these techniques [21].

Furthermore, in our cohort, SDD was achieved following RASP, whereas the majority of HoLEP patients stayed overnight (*p* < 0.001). Likewise, Palacios et al. in their study comparing the outcomes of SP-RASP and HoLEP, reported comparable outcomes but more frequent SDD for SP-RASP [10]. In contrast, Zhang et al. reported shorter hospital stays following HoLEP [9], which could be attributed to variations in surgeon-specific practices and clinical workflows. For instance, postoperative management following HoLEP has evolved in some institutions, making SDD standard practice [18]. We attribute our high rates of SDD following RASP to the urethral mucosa anastomosis, which eliminates the need for postoperative CBI, and the robotic TAP block at the conclusion of the case, which assists with improved pain control. On the other hand, at our institution, it is standard care for all HoLEP patients to remain admitted overnight for CBI and be discharged after a voiding trial on postoperative day 1.

To clarify, the purpose of this study is not to demonstrate the superiority of one technique over the other. Rather, we aim to show that both RASP and HoLEP are comparable in safety and efficacy, yet they offer distinct perioperative recovery courses. Patients need to be adequately counseled on expectations regarding the postoperative risks of tSUI, the duration of catheterization, CBI, and the length of hospital stay following the procedure. Additionally, factors such as urethral length, the presence of bladder stones, prior transabdominal surgery, costs, and availability of surgical equipment should be considered when advising patients on treatment options.

Finally, our study has limitations, including being conducted at a single tertiary care center with each type of surgery performed by a single, high-volume surgeon. Similar to the aforementioned studies, the generalizability of our findings is limited due to significant variations in surgical techniques and perioperative management among surgeons. Both surgeons in our study have over 20 years of experience, which likely impacts surgical outcomes such as operative time, blood loss, and postoperative tSUI. Further long-term follow-up is needed to assess the durability of these procedures. Although patient satisfaction metrics are arguably the most important postoperative outcome, we did not have standardized records and were unable to report on these measures. A prior study using a third-party-administered survey showed that HoLEP had the highest patient-reported satisfaction outcomes compared to other BPH interventions; however, this was a single-institution study that did not include RASP patients [22]. The higher costs of robotic surgery are well documented. Cost-effectiveness analysis has shown that HoLEP is the most cost-effective treatment option, however, this study did not include RASP in their analyses either [23]. Additional endpoints such as perioperative anticoagulant and/or antiplatelet use, opioid use, and postoperative pain, which are also critical outcomes, are not reported in our study. Further research is warranted to compare patient-reported satisfaction outcomes, cost-effectiveness, and postoperative pain and opioid use between matched cohorts of RASP and HoLEP. Finally, a major limitation of our study is the small sample size of 31 patients in each group following the propensity score matching, which can limit the generalizability of this study. Nonetheless, this is the first PSM-based analysis comparing RASP with HoLEP by two experienced surgeons, demonstrating comparable and favorable outcomes.

## 5. Conclusions

Our study suggests that RASP with bladder neck sparing technique and HoLEP with early apical release have comparable and favorable perioperative outcomes. There was no difference in tSUI rates between RASP and HoLEP. RASP requires a longer post-operative catheter time while providing a shorter hospital stay. Differences in surgeons’ techniques and surgeon-specific standard practices will impact the outcomes following these two surgeries.

## Figures and Tables

**Table 1:** Pre-, Peri-, and Postoperative Characteristics of the Cohorts before PSM.

Variable	HoLEP (n = 156)	RASP (n = 36)	*p* Value
Age, years, mean ± SD n_1_ = 156; n_2_ = 36	72.93 ± 8.82	70.86 ± 8.2	0.2
BMI, kg/m^2^, median (IQR) n_1_ = 156; n_2_ = 36	26.1 (24.1–30.2)	28.4 (24.7–31)	0.136
Previous interventions for enlarged prostate, n (%) n_1_ = 156; n_2_ = 36			
Yes	42 (26.9%)	7 (19.4%)	0.404
No	114 (73.1%)	29 (80.6%)	
Preop use of alpha-blockers, n (%) n_1_ = 156; n_2_ = 36			
Yes	114 (73.1%)	27 (75%)	0.839
No	42 (26.9%)	9 (25%)	
Preop use of 5 ARIs, n (%) n_1_ = 156; n_2_ = 36			
Yes	66 (42.3%)	17 (47.2%)	0.709
No	90 (57.7%)	19 (52.8%)	
Preop urinary retention, n (%) n_1_ = 156; n_2_ = 36			
Yes	82 (52.6%)	20 (55.6%)	0.853
No	74 (47.4%)	16 (44.4%)	
Preop AUASS, mean ± SD n_1_ = 44; n_2_ = 22	17.8 ± 7.6	21.2 ± 7.3	0.086
Prostate size, mL, median (IQR) n_1_ = 125; n_2_ =36	84 (54.5–120)	141.5 (104–158)	**<0.001**
Preop PVR, mL, median (IQR) n_1_ = 87; n_2_ = 17	150 (50–350)	124 (28.5–454.5)	0.626
ASA score, n (%) n_1_ = 156; n_2_ = 36			
≤2	81 (51.9%)	18 (50%)	0.855
≥3	75 (48.1%)	18 (50%)	
Operation time, minutes, median (IQR) n_1_ = 156; n_2_ = 36	109.5 (77–134)	131 (116.25–144.25)	**<0.001**
EBL, mL, median (IQR) n_1_ = 156; n_2_ = 36	25 (20–50)	100 (50–100)	**<0.001**
Perioperative complications, n (%) n_1_ = 156; n_2_ = 36	0	0	-
Length of hospital stay, days, median (IQR) n_1_ = 156; n_2_ = 36	1 (0)	0 (0)	**<0.001**
Postop complications, n (%) n_1_ = 156; n_2_ = 36	22 (14.1%)	4 (11.1%)	0.790
Clavien-Dindo Grade, n (%) ≥III	7 (4.5%)	3 (8.3%)	0.401
Postop PSA, ng/mL, median (IQR) n_1_ = 84; n_2_ = 28	0.56 (0.3–1.32)	0.8 (0.44–1.89)	0.095
Length of catheterization, days, median (IQR) n_1_ = 156; n_2_ = 36	1 (1–4)	7 (7–8)	**<0.001**
Readmission in 30 days, n (%) n_1_ = 156; n_2_ = 36	5 (3.2%)	2 (5.6%)	0.617
Readmission in 90 days, n (%) n_1_ = 156; n_2_ = 36	6 (3.8%)	4 (11.1%)	0.094
Pathology, n (%): n_1_ = 156; n_2_ = 36			
Benign	128 (82.1%)	34 (94.4%)	0.076
Cancer	28 (17.9%)	2 (5.6%)	
*Incidental prostate cancer*	22 (14.8%)	1 (2.9%)	0.084
Pathological weight of resected adenoma, grams, median (IQR) n_1_ = 156; n_2_ = 36	40 (15.5–71.2)	72.8 (56.1–99.2)	**<0.001**
Postop PVR, mL, n (%): n_1_ = 87; n_2_ = 12			
≤50	61 (70.1%)	11 (91.7%)	0.171
>50	26 (29.9%)	1 (8.3%)	
Postop 3-month AUASS, n (%): n_1_ = 32; n_2_ = 21			
≤7	30 (93.8%)	13 (72.2%)	0.083
≥8	2 (6.3%)	5 (27.8%)	
SUI at postop 3 months, n (%): n_1_ = 149; n_2_ = 29			
Yes	8 (5.4%)	2 (6.9%)	0.668
No	141 (94.6%)	27 (93.1%)	
***Data available from: n*_1_ -> *HoLEP; n*_2_ -> *RASP***			

***Abbreviations***: SD—Standard Deviation, BMI—Body Mass Index, IQR—Interquartile Range, 5 ARIs—5-alpha Reductase Inhibitors, AUASS—American Urological Association Symptom Score, PVR—Postvoid Residual Volume, ASA—American Society of Anesthesiologists Physical Status Classification System, EBL—Estimated Blood Loss, PSA—Prostate Specific Antigen, SUI—Stress Urinary Incontinence.

**Table 2:** Pre-, Peri-, and Postoperative Characteristics of the Cohorts after PSM.

Variable	HoLEP (n = 31)	RASP (n = 31)	*p* Value
Age, years, mean ± SD n_1_ = 31; n_2_ = 31	70 ± 8.1	71 ± 8.2	0.385
BMI, kg/m^2^, median (IQR) n_1_ = 31; n_2_ = 31	26.1 (24.5–28.8)	28.4 (24.4–30.9)	0.360
Previous interventions for enlarged prostate, n (%) n_1_ = 31; n_2_ = 31	8 (25.8%)	6 (19.4%)	0.762
Preop use of alpha-blockers, n (%) n_1_ = 31; n_2_ = 31	21 (67.7%)	23 (74.2%)	0.780
Preop use of 5 ARIs, n (%) n_1_ = 31; n_2_ = 31	9 (29%)	15 (48.4%)	0.192
Preop urinary retention, n (%) n_1_ = 31; n_2_ = 31	18 (58.1%)	16 (51.6%)	0.799
Preop AUASS, mean ± SD n_1_ = 12; n_2_ = 19	21 ± 9	20 ± 6	0.707
Prostate size, ml, mean ± SD n_1_ = 31; n_2_ = 31	117 (95–160)	134 (103–154)	0.335
Preop PVR, mL, median (IQR) n_1_ = 17; n_2_ = 14	109 (68–169)	287 (88–457)	0.186
ASA score, n (%) n_1_ = 31; n_2_ = 31			
≤2	23 (74.2%)	15 (48.4%)	0.037
≥2	8 (25.8%)	16 (51.6%)	
Operation time, minutes, mean ± SD n_1_ = 31; n_2_ = 31	139 ± 34	128 ± 23	0.140
EBL, mL, median (IQR) n_1_ = 31; n_2_ = 31	40 (20–50)	100 (50–100)	**<0.001**
Perioperative complications, n (%) n_1_ = 31; n_2_ = 31	0	0	-
Length of hospital stay, days, median (IQR) n_1_ = 31; n_2_ = 31	1 (1–2)	0 (0)	**<0.001**
Postop complications, n (%) n_1_ = 31; n_2_ = 31	5 (16.1%)	3 (9.7%)	0.707
Postop PSA, ng/mL, median (IQR) n_1_ = 13; n_2_ = 25	0.8 (0.5–1.53)	0.8 (0.45–2.03)	0.879
Length of catheterization, days, median (IQR) n_1_ = 31; n_2_ = 31	1 (1–4)	7 (7–8)	**<0.001**
Readmission in 30 days, n (%) n_1_ = 31; n_2_ = 31	3 (9.7%)	2 (6.5%)	1
Readmission in 90 days, n (%) n_1_ = 31; n_2_ = 31	3 (9.7%)	4 (12.9%)	1
Pathology, n (%): n_1_ = 31; n_2_ = 31			
Benign	26 (83.9%)	29 (93.5%)	0.425
Cancer	5 (16.1%)	2 (6.5%)	
*Incidental prostate cancer*	4 (13.3%)	1 (3.3%)	0.353
Pathological weight of resected adenoma, grams, mean ± SD n_1_ = 31; n_2_ = 31	78.5 ± 36.4	70.9 ± 32	0.394
Postop PVR, mL, n (%): n_1_ = 19; n_2_ = 11			
≤50	13 (68.4%)	10 (90.9%)	0.215
>50	6 (31.6%)	1 (9.1)	
Postop 3-month AUASS, n (%): n_1_ = 18; n_2_ = 18			
≤7	17 (94%)	14 (78%)	0.338
≥8	1 (6%)	4 (22%)	
SUI at postop 3 months, n (%): n_1_ = 28; n_2_ = 25			
Yes	2 (7%)	2 (8%)	1
No	26 (93%)	23 (92%)	
***Data available from: n*_1_ -> *HoLEP; n*_2_ -> *RASP***			

***Abbreviations***: SD—Standard Deviation, BMI—Body Mass Index, IQR—Interquartile Range, 5 ARIs—5-alpha Reductase Inhibitors, AUASS—American Urological Association Symptom Score, PVR—Postvoid Residual Volume, ASA—American Society of Anesthesiologists Physical Status Classification System, EBL—Estimated Blood Loss, PSA—Prostate Specific Antigen, SUI—Stress Urinary Incontinence.

**Table 3:** The Details of Hospital Readmissions.

Variable	HoLEP (n = 31)	RASP (n = 31)	*p* Value
Readmission in 90 days, n (%) n_1_ = 31; n_2_ = 31	3 (9.7%)	4 (12.9%)	1
Reasons for readmission	Two patients with gross hematuria and clot retention. UTI requiring IV antibiotics.	Infected pelvic hematoma after a fall. Gross hematuria and clot retention after vigorous exercise 3 weeks postop. UTI and sepsis in a patient with a history of MRSA and ESBL-producing *E. coli* infections. Possible SBO that resolved with conservative management.	

***Abbreviations***: SBO—Small Bowel Obstruction, UTI—Urinary Tract Infection, IV—Intravenous, MRSA—Methicillin-resistant Staphylococcus aureus, ESBL—Extended-spectrum Beta-lactamase.

**Table 4:** Predictive Models for Pre- and Post-PSM Outcomes Based on Treatment (Constant -> RASP).

**Pre-PSM Outcomes**	**OR**	**95% CI**	***p*-Value**
AUASS at 3 months	0.173	0.03–1.01	0.052
SUI rates at 3 months	0.77	0.15–3.8	0.744
Complication rates	1.31	0.42–4.1	0.637
**Post-PSM Outcomes**	**OR**	**95% CI**	***p*-Value**
AUASS at 3 months	0.235	0.022–2.54	0.124
SUI rates at 3 months	0.89	0.12–6.79	0.906
Complication rates	1.8	0.39–8.277	0.453

***Abbreviations***: PSM—Propensity Score Matching, AUASS—American Urological Association Symptom Score, SUI—Stress Urinary Incontinence, OR—Odds Ratio, CI—Confidence Interval.

## Data Availability

The raw data supporting the conclusions of this article will be made available by the authors upon reasonable request.
